# Association between exposure to tobacco information through mass media, smoking households and secondhand smoke exposure in adolescents: Survey data from South Korea

**DOI:** 10.18332/tid/175705

**Published:** 2024-01-05

**Authors:** Wenbin Du, Gaoran Chen, Minmin Gu, Huixin Deng, Won G. Cho

**Affiliations:** 1 Research Institute of Social Development, Southwestern University of Finance and Economics, Chengdu, China; 2 SWUFE-UD Institute of Data Science, Southwestern University of Finance and Economics-University of Delaware, Chengdu, China; 3 Department of Social Welfare, College of Social Sciences, Jeonbuk National University, Jeonju, Korea

**Keywords:** secondhand smoke exposure, smoking households, tobacco information, mass media

## Abstract

**INTRODUCTION:**

To explore the current situation of exposure of Korean adolescents to secondhand smoke (SHS) in households our study aimed to determine the relationship between family member smoking status, exposure to tobacco information through mass media, and household SHS exposure.

**METHODS:**

The present study uses pooled data from the Korean adolescent health behavior online survey conducted in 2015, 2018 and 2021, with 157944 participants. The regression models were used to explore the association between the smoking status of households, and exposure to tobacco information through mass media, and household SHS exposure in adolescents, controlling for potential confounding factors.

**RESULTS:**

SHS exposure duration of Korean adolescents in households was 0.88 days per week. The households with smokers including the father (β=1.087; 95% CI: 1.0–1.126), mother (β=1.461; 95% CI: 1.379– 1.543), siblings (β=0.545; 95% CI: 0.493–0.597), grandparents (β=0.224; 95% CI: 0.174–0.272), and other relatives (β=0.170; 95% CI: 0.126–0.214), showed a positive association with SHS exposure in adolescents within the household. At the same time, information about anti-smoking ads on television (β= -0.042; 95% CI: -0.069 – -0.015) and public transportation (β= -0.031; 95% CI: -0.054 – -0.010), showed a negative association with SHS exposure in adolescents. However, broadcasts, online, and newspaper non-smoking ads were not associated with SHS exposure (p>0.05). In addition, regression models revealed that exposure to cigarette advertising in magazines (β=0.131; 95% CI: 0.097–0.166), networks (β=0.151; 95% CI: 0.127–0.175), convenience stores (β=0.061; 95% CI: 0.035–0.087), and supermarkets (β=0.133; 95% CI: 0.108–0.158) is associated with SHS exposure in adolescents. Finally, our study showed stronger ties between SHS exposure, family smoking, and tobacco ads in girls. The link between maternal smoking, supermarket ads, and adolescent SHS exposure intensified in 2021 compared to 2015.

**CONCLUSIONS:**

Family and media were identified as potential factors associated with SHS exposure in adolescents. Therefore, publicity and education regarding household SHS hazards, and smoking bans in media, can be helpful in protecting adolescents from SHS.

## INTRODUCTION

Secondhand smoke (SHS), also known as passive or involuntary smoking, usually refers to the inhalation of tobacco smoke or smoke from cigarette burning by non-smokers against their will^1^. Exposure to SHS is a major health problem in adolescence^2^. Overall, 40% of children and teenagers worldwide are exposed to SHS, a major indoor pollutant^3^. Passive exposure to tobacco smoke poses a serious risk to children and teenagers, as it may cause respiratory diseases and adversely affect the development and maturation of the lungs, as well as cardiovascular and brain functions^4^, even increasing the risk of several cancers^5^. Adolescents are in the developmental stage and thus are more vulnerable to physical damage due to their weak resistance to external harmful factors and sensitivity to the harm caused by smoke. SHS exposure also affects students’ cognitive and learning abilities^6^. In addition, SHS exposure increases the risk of smoking attempts among middle school students^7^.

After formulating the National Health Promotion Act in 1995, South Korea expanded its policy to reduce SHS exposure^8^. Furthermore, since 1998, South Korea has been implementing a smoking cessation program with a strong emphasis on anti-smoking campaigns and education regarding the hazards of secondhand smoke. Starting in 2002, this initiative extended to junior high schools and high schools, led by municipal and provincial government offices, aiming to implement ‘Adolescent Smoking Prevention and Secondhand Smoke Hazard Education’. Subsequently, the WHO Framework Convention on Tobacco Control, established in 2005, has implemented the no-smoking policies in South Korea. The 2020 Korea Community Health Survey showed a continued decline in SHS exposure in Korea. However, despite the government’s efforts, the 16th Korea Adolescent Health Behavior Survey conducted in 2020 found that the SHS exposure rate in adolescent households was 20.0% for boys and 22.4% for girls. These rates were still higher than those in other countries^9,10^. The data revealed that adolescent SHS exposure has relatively complicated causes and needs more attention and research.

Home is among the most crucial places for teenagers’ daily activities and is where teenagers are most exposed to SHS^11^. Children whose parents smoke consider smoking a normal behavior, and therefore, they are more likely to become smokers^7^. SHS exposure increases the possibility that non-smoking teenagers will have their first try and start developing the pernicious habit of smoking^12^. In traditional Korean families, cohabitation often spans three to four generations, while many contemporary families primarily comprise couples. The influence of various family members, particularly in multi-generational households, on non-smoking adolescents has not been fully explored. Reducing household SHS exposure can effectively reduce the adverse health effects of passive exposure to tobacco on adolescents^13,14^. Therefore, to protect adolescents from SHS, an effective response to family smoking, propaganda, and education for preventing SHS is extremely urgent. To address this concern, our study considered non-smokers as subjects for analyzing the influence of different family members on the SHS exposure rate in adolescents and the current situation of SHS exposure in adolescents, to provide a basis for policymakers and executors. The findings can be used to determine how to effectively promote anti-smoking education in the family.

Public awareness of the harm caused by cigarettes and SHS has recently increased among young people in South Korea. Most people learn about these dangers through television (TV), the Internet, and advertisements at subway stations^15^. Previous studies have shown that exposure to tobacco-specific information through advertising, media, and interpersonal sources may influence a person’s attitudes toward the dangers of tobacco use, the decision to smoke or quit, or the support for tobacco control policies^16-18^. South Korea began to launch anti-smoking projects focused on anti-smoking publicity and education in 1998. Since 2002, the South Korean government has implemented ‘Smoking Prevention and Smoking Prohibition Education for Teenagers’ in junior and senior high schools, centering on the city and province departments of education^19^. Since 2000, smoking ban advertisements have been produced yearly and broadcasted through TV, radio, theatre, subway, and other media. The government has also started anti-smoking-related programs to create a non-smoking atmosphere^19^. Using mass media to prevent smoking and enhance support of the smoking ban may lead to negative attitudes toward smoking, suggesting that mass media are effective in promoting smoking cessation^20,21^. In South Korea, although the focus on the use of mass media for tobacco hazard and anti-smoking advertising has gradually increased recently, only a few studies have investigated the association between SHS exposure and tobacco-specific information (exposure to tobacco advertising and anti-smoking advertising) from mass media. Therefore, clarifying the correlation between tobacco advertising, anti-smoking advertising, and SHS exposure in Korean adolescents through systematic research is necessary. Our study targets Korean adolescents who are exposed to various tobacco advertising and anti-smoking advertising media to investigate the correlation of exposure to tobacco-specific information from mass media with adolescents’ exposure to SHS. Additionally, this research explores the association between public and home environments and secondhand smoke exposure among Korean adolescents. The term ‘public environment’ encompasses the exposure of adolescents to tobacco information through mass media in public places such as public transportation, whereas the ‘home environment’ pertains to their exposure to secondhand smoke within the household. This differentiation is crucial for understanding the associations between environments and secondhand smoke exposure.

## METHODS

### Data sources and study sample

Our study is based on the secondary dataset analyses of the Korea Youth Risk Behavior Web-based Survey (KYRBWS), an annual survey conducted by the Department of Disease Management since 2005. Students from grade 1 of middle school to grade 3 of high school have to complete this anonymous online survey. Based on April 2021 data, the national junior and senior high school students were considered to be the target population. Sampling was conducted on the target population stratification, sample allocation, and sample staging. We used 39 regional groups and education levels (junior high school, general high school, and special high school) as stratification variables in the single-level admission stage. The sample was divided into 117 levels. In sampling distribution, the samples were allocated to 400 middle and 400 high schools, and the proportional distribution was used to allocate the number of sample schools. Sampling was performed according to the hierarchical community extraction method. The school was considered the extraction unit for the first time, whereas the class was the extraction unit for the second time^22^. From 2005 to 2021, the KYRBWS conducted 21 waves of data collection. However, only three of these waves contained the complete research variables required for our study. To maintain consistency across variables and explore the temporal trends in exposure to tobacco-related information, we opted to work with three-year samples with uniform variables. These samples were taken from KYRBS 2015 (n=68043), KYRBS 2018 (n= 60040), and KYRBS 2021 (n=54848). We then pooled these data into an independent, mixed cross-sectional dataset. After dealing with missing values, 157944 samples were included in the study.

### Outcome variable

*Household SHS exposure*

To assess SHS exposure among Korean adolescents at home, they were asked the following question ‘How many days have you been exposed to secondhand smoke at home in the last seven days?’. Here, secondhand smoke includes emissions from ordinary cigarettes, liquid e-cigarettes, and nicotine-containing e-cigarettes. Accordingly, the participants were explicitly instructed that ‘secondhand smoke’ refers to smoke from both traditional cigarettes and e-cigarettes, and were given the following options: no exposure in the last seven days, exposure for one day, exposure for two days, exposure for three days, exposure for four days, exposure for five days, exposure for six days, and exposure for seven days.

### Predictive variables

*Exposure to tobacco information through mass media*

To assess the exposure of Korean adolescents to tobacco information through mass media, they were asked the following two questions: ‘Have you seen or heard the following propaganda of the smoking ban in the past 12 months? and ‘Have you seen cigarette advertisements in the last 30 days?’. For the first question, the participants could provide the following responses: never seen, TV anti-smoking ads, radio anti-smoking ads, Internet anti-smoking campaigns, and newspaper articles and advertisements. For the second question, they could provide the following responses: no, magazines, Internet, convenience stores, and supermarkets. Two separate binary variables were created to represent exposure to anti-smoking media (coded as 1 for exposure and 0 for no exposure) and exposure to pro-smoking media (coded as 1 for exposure and 0 for no exposure).

*Smoking households*

A smoking household was defined as any family with a smoker, that is, ‘people in the household who smoke now’ The adolescents were asked: ‘Who in the household smokes now?’. Response choices were: no, father, mother, siblings, grandparents, and other relatives. This question was used to measure the smoking status of households of adolescents. For each category of family member exhibiting smoking behavior, the corresponding variable was assigned the value 1, otherwise the value 0. Meanwhile, the variable of ‘no family member smoking’ was used to distinguish between smoking and non-smoking families and determine the different SHS exposure situations among families of adolescents.

### Controlled variables

We constructed a set of covariates that may affect household exposure of adolescents to SHS. These covariates were gender, school type, school level, city size, residence, family socioeconomic status (on a scale of 1–5, the higher the score, the better the family socioeconomic status), father’s education level, and mother’s education level.

### Statistical analyses

First, we conducted a descriptive analysis to outline all variables. Next, we utilized the R package *doBy* to perform paired t-tests, report significance levels after two-tailed tests, and visualize the t-test results using the *ggplot2* package. Finally, we employed ordinary least squares (OLS) regression models to analyze the impact of exposure to tobacco-related information through mass media, and smoking household, on secondhand smoke (SHS) exposure in the adolescent’s household. We also tested the moderating effects of gender and survey year. The data used in this study comprises three waves of data, each from 800 schools. This results in a challenge of heteroskedasticity in the data and non-independence of observations. To address this issue and capture the true variability of the estimated coefficients, we adjusted the standard errors. We employed cluster robust standard errors (CRSEs) as an effective method^23^. We used the *lm_robust* function in the R package *estimatr* while using survey schools as the clustering variables.

## RESULTS

### Descriptive statistical analysis

Table 1 provides a statistical description of the study variables. Boys and girls accounted for 51.5% and 48.5% of the total sample, respectively. Middle and high school students accounted for 48.3% and 51.7%, respectively. The score of SHS exposure of the sample adolescents in 7 days was between 0 and 7, with a mean value of 0.88. The mean value of SHS exposure suggests that, on average, adolescents were exposed to SHS for nearly one day out of seven. This level of exposure is slightly lower than the global average for adolescent SHS exposure^24^. Overall, 95% of Korean adolescents live with their families, which implies that they are at a high risk of exposure to SHS originating from their family members. Among all family members, the smoking rate of fathers was the highest at 42.8%. The smoking rates of mothers, siblings, grandparents, and other family members were 3.3%, 5.5%, 6.3%, and 6.7%, respectively. The proportion of adolescents who reported having heard or seen anti-smoking advertisements on TV, radio, news, Internet, newspapers, and public transportation in the past 12 months was 62.9%, 10.4%, 24.8%, 34%, 18.2%, and 16.8%, respectively. Thus, teenagers are most exposed to anti-smoking advertisements on TV. In addition, the proportion of adolescents who had seen cigarette advertisements in magazines, Internet, convenience stores, and supermarkets in the past 30 days was 9.5%, 38.2%, 53.8%, and 74.9%, respectively. In Korea, most cigarettes are sold in convenience stores and supermarkets, and our analysis revealed that teenagers are most exposed to cigarette advertisements at these places. Other characteristics of the adolescent sample are summarized in Table 1.

**Table 1:** Descriptive statistics for all variables, South Korea (2015, 2018, 2021) (N=157944)

*Variables*	*%*
**House secondhand smoke exposure**, mean ± SD (range)	0.88 ± 1.87 (0–7)
**Gender**	
Male	51.5
Female	48.5
**Family socioeconomic status**, mean ± SD (range)	2.68 ± 0.9 (1–5)
**Residence**	
Living with family	95.0
Staying with relatives	0.7
Living alone	1.6
Live in a dormitory	2.4
Residential childcare facilities	0.2
**School type**	
Middle school	51.7
High school	48.3
**Non-smoking ads exposure**	
Non-smoking ad (Ref. no)	23.0
TV (Ref. no)	62.9
Broadcasts (Ref. no)	10.4
Online (Ref. no)	34.0
Newspapers (Ref. no)	18.2
Public transportation (Ref. no)	16.8
**Smoking ads exposure**	
Magazines (Ref. no)	9.5
Online (Ref. no)	38.2
Convenience stores (Ref. no)	53.8
Supermarkets (Ref. no)	74.9
**Family smoking status**	
No smokers (Ref. no)	15.2
Father smokes (Ref. no)	42.8
Mother smokes (Ref. no)	3.3
Siblings smoke (Ref. no)	5.5
Grandparent smokes (Ref. no)	6.3
Other relatives smoke (Ref. no)	6.7
**City scale**	
County district	7.8
Big city	44.0
Small- and medium-sized city	48.2
**School type**	
Co-educational	59.2
Boys school	23.9
Girls school	16.9
**Father’s education level**	
Junior middle school	2.0
Senior middle school	26.0
University	18.5
Other	18.5
**Mother’s education level**	
Junior middle school	1.7
Senior middle school	31.3
University	49.6
Other	17.4

**Figure 1:**
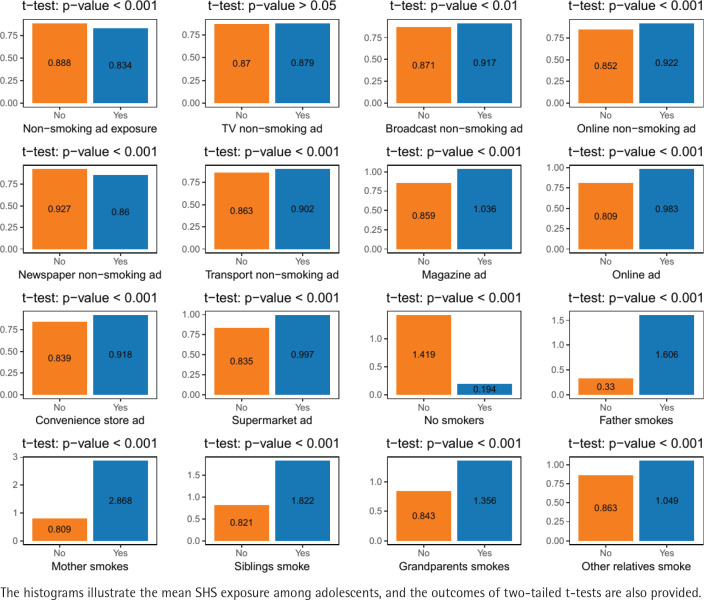
Paired t-test results for the differences SHS exposure between adolescents with and without mass media and smoking household exposure, South Korea (2015, 2018, 2021) (N=157944)

The effects of exposure to tobacco information through mass media and the smoking status of family members were compared between adolescents with and without SHS exposure. Figure 1 shows statistically significant differences between the two groups in terms of exposure to radio, news, Internet, newspapers, and public transportation non-smoking ads; exposure to cigarette advertising in magazines, Internet, convenience stores, and supermarkets; and the smoking status of all family members (p<0.05).

To further analyze the factors influencing SHS exposure in Korean adolescents, two models were constructed by using SHS exposure in the family as the outcome variable; exposure to tobacco information through mass media and family members who smoke as predictor variables; and school type, gender, grade, socioeconomic status, city scale, parents’ education level, and other demographic characteristics as covariates. Model 1 was used to explore the association between exposure to tobacco information through mass media and household SHS exposure in adolescents and to control potential confounding factors. Table 2 shows that TV and public transportation non-smoking ads are negatively correlated with household SHS exposure in adolescents, indicating that these two variables are protective factors for SHS exposure in adolescents in households. The regression coefficient of TV non-smoking ads to household SHS exposure was -0.042 (95% CI: -0.069 – -0.015, p<0.01). The regression coefficient of public transport non-smoking ads to household SHS exposure was -0.031 (95% CI: -0.054 – -0.010, p<0.01). However, the association between SHS exposure and non-smoking ads on radio, online, and in newspapers, was not statistically significant (p>0.05). There was a positive correlation between non-smoking ads in the news and SHS exposure (β=0.072; 95% CI: 0.048–0.096, p<0.001). It is important to note that this does not imply that news non-smoking ads directly caused an increase in SHS exposure among adolescents. Instead, this association could be attributed to the Korean government’s efforts to intensify non-smoking advertisements in regions where adolescent SHS exposure was particularly high. Finally, cigarette advertisements in magazines (β=0.131; 95% CI: 0.097–0.166, p<0.001), online (β=0.151; 95% CI: 0.127–0.175, p<0.001), convenience stores (β=0.061; 95% CI: 0.035–0.087, p<0.001), and supermarkets (β=0.133; 95% CI: 0.108–0.158, p<0.001), were positively correlated with SHS exposure in households and thus are the risk factors for household SHS exposure in adolescents.

**Table 2:** Regression results, South Korea (2015, 2018, 2021) (N=157944)

*Variables*	*β*	*p*	*95% CI*
**Model 1 Public environment**			
**Non-smoking ads exposure**			
No ad exposure	0.01	0.646	-0.026–0.042
TV	-0.042	0.003	-0.069 – -0.015
Broadcasts	0.011	0.509	-0.021–0.0423
News	0.072	<0.001	0.048–0.096
Online	0.001	0.927	-0.022–0.024
Newspapers	-0.016	0.243	-0.042–0.011
Public transportation	-0.031	0.005	-0.054 – -0.010
**Smoking ads exposure**			
Magazines	0.131	<0.001	0.097–0.166
Online	0.151	<0.001	0.127–0.175
Convenience stores	0.061	<0.001	0.035–0.087
Supermarkets	0.133	<0.001	0.108–0.158
**Control variables**			
Intercept	1.180	<0.001	1.039–1.322
R^2^	0.031		
**Model 2 Family environment**			
**Family smoking status**			
No smokers	-0.148	<0.001	-0.185 – -0.111
Father smokes	1.087	<0.001	1.049–1.126
Mother smokes	1.461	<0.001	1.379–1.543
Siblings smoke	0.545	<0.001	0.493–0.597
Grandparent smokes	0.224	<0.001	0.174–0.272
Other relatives smoke	0.170	<0.001	0.126–0.214
**Control variables**			
Intercept	0.764	<0.001	0.636–0.892
R^2^	0.166		

In the two models: gender, family socioeconomic status, residence, father’s education level, mother’s education level, and survey years, were statistically controlled.

Model 2 was used to explore the association between smoking family factors (family members who smoke) and household SHS exposure and to control potential confounding factors. Table 2 shows that after controlling other factors by the model, the correlation between family members who smoke and household SHS exposure was positive and significant (p<0.001). Compared with other family members, smoking by mothers exerted a more substantial influence on adolescent SHS exposure (β=1.461; 95% CI: 1.379–1.543, p<0.001). These results indicate that smoking by family members including fathers, mothers, siblings, grandparents, and other relatives, is a risk factor for household SHS exposure in adolescents.

**Figure 2:**
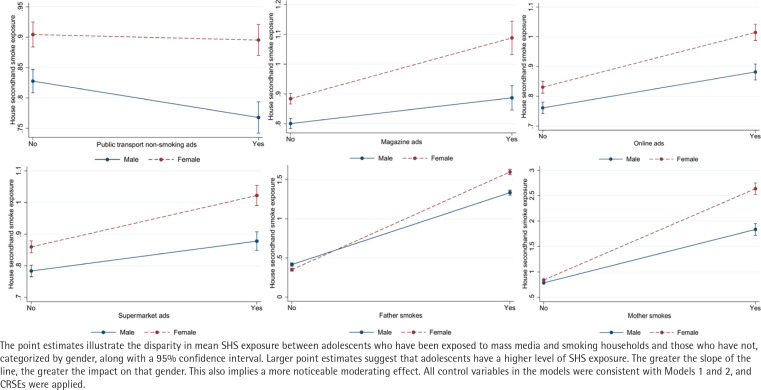
Moderating effect of gender, South Korea (2015, 2018, 2021) (N=157944)

**Figure 3:**
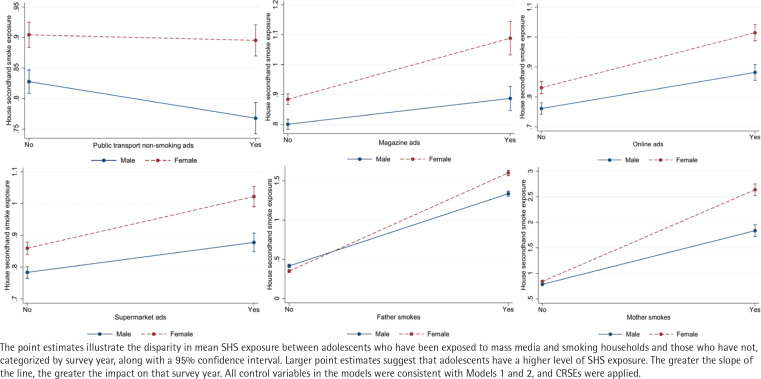
Moderating effect of survey time, South Korea (2015, 2018, 2021) (N=157944)

To examine the relationship between exposure to tobacco information through mass media and smoking households and secondhand smoke exposure in adolescents across different genders and survey years, this study developed 34 regression models to assess the moderating effects of gender and survey year. Due to the considerable number of parameters in these 34 regression models, we have presented and visualized the outcomes of 12 models exhibiting significant moderating effects (Figures 2 and 3).

Figure 2 shows that the adverse impact of transport non-smoking ad settings on SHS exposure was more pronounced in male adolescents. Furthermore, regarding SHS exposure, female adolescents appear to be more influenced by smoking ads in supermarkets, on the Internet, and in magazines compared to their male counterparts. Additionally, the smoking habits of both fathers and mothers have a greater effect on SHS exposure among female adolescents than among their male counterparts.

As shown in Figure 3, there are four noteworthy trends to highlight. First, the impact of supermarket ads on adolescent SHS exposure has increased in 2021 compared to 2015. Second, the adverse effect of non-smoking individuals and family members on adolescent SHS exposure weakened in 2018 and 2021, compared to 2015. Third, the influence of paternal smoking, grandparent smoking, and smoking by other relatives on adolescent SHS exposure decreased in 2021 compared to 2015. Fourth, the impact of maternal smoking on adolescent SHS exposure, on the other hand, has strengthened in 2021 compared to 2015. These trends suggest that both supermarket ads and maternal smoking warrant more attention in the context of adolescent SHS exposure.

## DISCUSSION

SHS exposure is a global public health concern and also a crucial health problem in adolescents. Attention should be paid to the problem of SHS exposure among Korean adolescents. Our study showed that the duration of SHS exposure among Korean teenagers in households was 0.88 days, reflecting that their SHS exposure was not high, consistent with previous study results. The relative importance of household SHS exposure is increasing because South Korea and other high-income countries have gradually adopted tobacco control laws and related regulations^25^. If smoking restrictions are strengthened, SHS exposure can be reduced in schools, public institutions, families, workplaces, etc. Considering the United States as an example, the Public Housing Authority has implemented a partial or total no-smoking policy on all public housing since 2009^26^, increasing people’s awareness of smoking hazards in households. Korea’s non-smoking area policy was implemented in 1995 when the National Health Promotion Act was enacted, but it gradually expanded. From 2010, local autonomy groups in Korea could designate no-smoking areas and levy fines based on the regulations. Starting in 2016, local autonomy groups could designate non-smoking areas and smoking places in cooperative housing societies such as apartments. At the same time, smoking and anti-smoking policies are gradually being promoted and expanded. Therefore, South Korea’s anti-smoking policy may have a positive effect on household SHS exposure.

The study analyzed the association between family members in Korean families and SHS exposure in adolescents using the OLS models. This analysis helped us determine the impact of smoking households (smoking status of family members) on adolescents’ SHS exposure. The study revealed that the smoking status of all family members was positively associated with household SHS exposure in adolescents, which is consistent with previous findings^15,16^. However, smoking by the mother had a stronger significant effect on household SHS exposure in adolescents than other family members’ smoking. This may be because most women in South Korea leave the labor market to become full-time housewives after childbirth^27^. Teenagers live longer with their mothers and are more likely to be exposed to SHS. Although the smoking habit of mothers has a greater impact on SHS exposure in adolescents, the smoking habit of fathers cannot be ignored. Thus, the smoking habits of all family members have a considerable impact on SHS exposure in adolescents. Based on the results mentioned above, to reduce exposure to cigarettes and improve environmental factors as much as possible, a non-smoking environment should be created in the living space, especially in the apartment balcony, corridors, and other indoor living spaces. Simultaneously, anti-smoking publicity campaigns or education for adolescents should cover families and communities rather than only individuals. This result further shows that changing cognition only from the individual level is insufficient. The government should strengthen public awareness and education about the harm caused by SHS to family members. It should formulate a complete anti-smoking policy in the space of family cohabitants or indoor areas of the community and effectively implement it to control the smoking rate and SHS exposure^28-30^.

Our study also analyzed the association between exposure of adolescents to tobacco information through mass media and SHS. Further analysis revealed that access to information from TV and public transportation non-smoking ads was negatively correlated with SHS exposure in adolescents.

However, broadcast, online, and newspaper non-smoking ads were not associated with SHS exposure. The lack of association between these different anti-smoking publicity ads and SHS exposure in adolescents may be due to the fact that past anti-smoking publicity ads were primarily run by non-profit organizations such as the Korean Anti-Smoking Campaign Association and the Public Service Advertising Association. However, because of the lack of expertise, uncertainty about the effect of anti-smoking advertising, and difficulty in ensuring funds, advocating for the necessity to quit smoking and the dangers of SHS exposure through media with credibility such as TV or public transportation ads, may be better than that through other mass media channels. The findings suggest that the association between different types of anti-smoking advertising exposure and SHS exposure in adolescents may influence public policies and research related to smoking bans or SHS exposure^31^. We should not only have campaigns on TV and public transportation, which have strong credibility, but also broadcast or plan programs related to the smoking ban and the harm of SHS, in various types of media such as online networks, social media, and automobile broadcasting^32^. In addition, the regression models revealed that exposure to cigarette advertising in magazines, networks, convenience stores, and supermarkets is a risk factor for SHS exposure in adolescents. Studies have shown that smoking images, trademarks, and ads displayed in public places through media such as TV, billboards, and newspapers, are risk factors for smoking behaviors^23^. Exposure to advertising increases the smoking behavior of people, thereby increasing the smoking exposure of those nearby.

### Limitations

Our study adopted an independent mixed cross-sectional dataset, so causality could not be determined. Subsequently, our study preliminarily explored the SHS exposure of Korean adolescents in households, warranting further exploration of the exposure of these adolescents to SHS in public places and schools. Additionally, response bias and recall bias, which might have influenced the study’s outcomes, are limitations that need to be addressed in future studies.

## CONCLUSIONS

Family and media may be the crucial factors influencing SHS exposure in adolescents, indicating the pivotal role of publicity and education of household SHS hazards, smoking bans, and the media in SHS exposure. Teenagers and family members can learn about the dangers of SHS through media publicity and thus make the right choices for their own and others’ health.

## Data Availability

The data supporting this research are available from the following link: https://www.kdca.go.kr/yhs/
