# Corrigendum: Association between exposure to tobacco information through mass media, smoking households and secondhand smoke exposure in adolescents: Survey data from South Korea

**DOI:** 10.18332/tid/178472

**Published:** 2024-01-16

**Authors:** Wenbin Du, Gaoran Chen, Minmin Gu, Huixin Deng, Won G. Choi

**Affiliations:** 1 Research Institute of Social Development, Southwestern University of Finance and Economics, Chengdu, China; 2 SWUFE-UD Institute of Data Science, Southwestern University of Finance and Economics-University of Delaware, Chengdu, China; 3 Department of Social Welfare, College of Social Sciences, Jeonbuk National University, Jeonju, Korea

**Keywords:** secondhand smoke exposure, smoking households, tobacco information, mass media

Corrigendum on:

Association between exposure to tobacco information through mass media, smoking households and secondhand smoke exposure in adolescents: Survey data from South Korea

By Wenbin Du^1^, Gaoran Chen^1^, Minmin Gu^1^, Huixin Deng^2^, Won G. Choi^3^

Tobacco Induced Diseases, Volume 22, Issue January, Page 1-11, Publish date: 5 January 2024

DOI: https://doi.org/10.18332/tid/175705

In the corrected version of the article, the last author’s surname was corrected from Cho to Choi. The mentioned change is corrected also online.

